# Treating status epilepticus in clinical practice—a multi-national survey in Germany, Austria, and Switzerland

**DOI:** 10.3389/fneur.2025.1685993

**Published:** 2025-11-03

**Authors:** Leona Möller, Urs Fisch, Lena Habermehl, Clara Jünemann

**Affiliations:** ^1^Philipps University Marburg, Department of Neurology, Epilepsy Center Hessen, Marburg, Germany; ^2^Department of Neurology, Brigham and Women’s Hospital, Harvard Medical School, Boston, MA, United States; ^3^Department of Neurology, University Hospital Basel, Basel, Switzerland; ^4^Epilepsy Center, Neurological Institute, University Hospitals Cleveland Medical Center, Cleveland, OH, United States

**Keywords:** epilepsy, status epilepticus, anti-seizure medication, neurocritical care, prehospital management, anesthetic agents

## Abstract

**Background:**

Status epilepticus (SE) is a life-threatening neurological emergency, and exhibits significant variability in clinical management despite established guidelines. This study evaluates current practices across German speaking countries.

**Methods:**

A web-based survey (December 2023–May 2024) assessed SE treatment strategies among 83 neurologists and neurointensivists from Germany, Austria, and Switzerland. Cases were presented to analyze diagnostic and therapeutic preferences.

**Results:**

The preferred benzodiazepine for first line treatment was lorazepam, chosen by 71.6% of the respondents. In the case of established SE, 35.4% chose levetiracetam as the preferred ASM. Propofol in combination with sufentanil/fentanyl was the preferred anesthetic of choice in 65.4% of respondents. For super-refractory status epilepticus (SRSE), 41.5% prefer to add further ASM, with valproic acid (67.1%), and lacosamide (64.5%) being the most frequently selected. Only 31.8% reported that their emergency services have a standard operating procedure (SOP) for SE treatment, and the choice of the preferred benzodiazepine varied in the preclinical setting, with midazolam being the most commonly used. 1) *First-line therapy*: Lorazepam (71.6% in-hospital), midazolam (50% prehospital), 2) *Second-line therapy*: Levetiracetam (35.4%) and lacosamide (13.4%) were the most common choices, 3) *Refractory SE*: Propofol with opioids (65.4%) were preferred for anesthesia, 4) *Prehospital care*: 31.8% of emergency services lacked standardized protocols; midazolam dosing varied widely (2–10 mg).

**Diagnostics:**

Laboratory testing was universal (96.9%), but MR-imaging (10%) and clinical use of prognostic scores (6.2%) were underutilized.

**Conclusion:**

This survey highlights the variability in clinical practice for managing status epilepticus in German-speaking countries. Persistent heterogeneity in SE management underscores the need for standardized protocols, particularly in prehospital care and refractory SE therapy.

## Introduction

With an incidence of 10–40 per 100,000 person-years and a 7%–33% mortality, status epilepticus (SE) is one of the most common life-threatening emergencies in neurology and is associated with significant mortality and morbidity (1, 2, 36).

Recommended by international guidelines, the management of SE follows a stepwise approach, starting with the administration of benzodiazepines, as first-line therapy. If SE persists, second-line treatment including anti-seizure medication (ASM) such as levetiracetam, valproate, and lacosamide, should be given within the first 30 min. In cases of refractory or super-refractory SE (SRSE), where seizures continue despite multiple treatments, deeper sedation with anesthetic agents like propofol and even more advanced interventions may be necessary (3–5). SRSE occurs in about 12% of all cases of SE (6). Kantanen et al. (7) identified 75 patients treated in the ICU and under anesthesia, corresponding to an annual incidence of 3.0. Of these, 21% were classified as SRSE, with the annual incidence being 0.6/100,000.

While guidelines advocate benzodiazepines followed by i.v.-ASM and anesthetics, real-world adherence to these guidelines remains inconsistent (8, 9).

Furthermore, new-onset refractory status epilepticus (NORSE), a SE characterised by the occurrence of a refractory SE in a patient without a prior history of epilepsy and without any obvious underlying etiology, poses a particular challenge for intensive care therapy (10). With a mortality rate of 12% and a high risk of survivors developing therapy-resistant epilepsy, the prognosis remains poor despite modern intensive care treatments. The survey also inquired about the usual diagnosis and treatment of NORSE.

To better understand different approaches to SE management in the German-speaking countries (Germany, Austria, Switzerland, DACH), a web-based survey on the treatment of SE was performed among clinicians focusing particularly on ASM choices and dosage and diagnostic procedures. The findings of this survey provide valuable insights into the current state of SE care, offering an opportunity to compare clinical practices and identifying potential areas for improvement in clinical guidelines and research.

## Methods

An anonymous web-based survey was conducted from December 2023 until May 2024 among neurologists in Germany, Austria and Switzerland (https://surveymonkey.de, SurveyMonkey Europe UC). The questionnaire consisted of two parts. The first part presented two case-based scenarios addressing escalating SE management from preclinical emergency care of early SE, to established SE, to SRSE. The second part included specific questions on SE management in the prehospital setting and diagnostic procedures. The survey was completed with voluntary demographic information provided by the respondents.

This case-based scenarios and structured questions on SE management was distributed via IGNITE and MuSE networks. The complete questionnaire is available in the Supplementary material. The survey was distributed via the IGNITE (Initiative for German NeuroIntensive Trial Engagement, a section of the German Society for Neurointensive and Emergency Care) and MuSE (Multicentric Studies in Epilepsy, part of the German, Austrian and Swiss epilepsy societies) networks. This is a network of young neurointensive care physicians and epileptologists who are always open to new members, so no specific number of members can be given. In addition, members had the opportunity to pass the survey on to colleagues.

The survey was distributed via email to participants of the different networks, followed by a reminder notification after 2 months.

Descriptive statistical analyses were performed using the SurveyMonkey and jamovi (v2.3.28) software.

## Results

A total of 83 clinicians (77.1% attendings/senior physicians, 68.8% university-affiliated, 52.5% EEG-certified, 32.8% board-certified intensivists, 18% board-certified in emergency medicine, 31.2% were epileptologists) completed the survey. A substantial proportion of respondents (68.8%) were affiliated with university hospitals, additional 28.1% reported working in specialized epilepsy centers, while only five participants indicated employment in the outpatient sector. This reflects the target audience of the networks contacted. Furthermore, the questionnaire was completed primarily by specialists and senior physicians (25% specialists, 57.8% senior physicians), which is consistent with the distribution within the networks. The first digit of the postcode was also requested in order to avoid individual areas being overrepresented. Fortunately, there were no significant differences here; all areas were covered. Subgroup analysis did not reveal major systematic differences between countries, although minor numerical trends were observed.

## Part 1: therapy escalation management based on a clinical scenario

### Case 1

A case describing SE in an early stage was presented first: “A 36-year-old patient (80 kg) is presented to the emergency department. He was given 5 mg diazepam preclinically for generalized convulsive status epilepticus (SE), which did not successfully treat the SE.”

**Figure 1:**
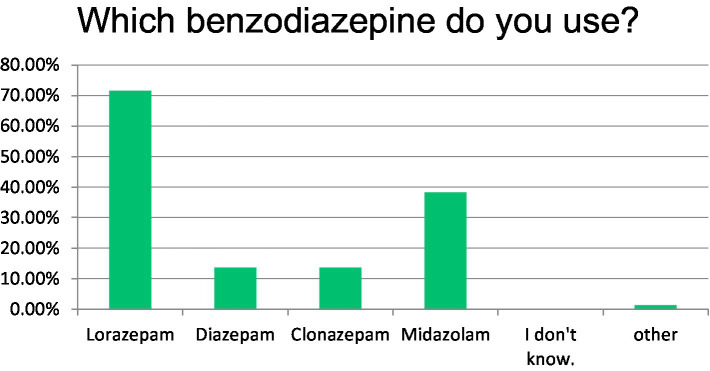
Benzodiazepine chosen for SE therapy stage 1.

In this presented clinical scenario of convulsive SE, 94% of clinicians administered additional intravenous benzodiazepines, with 57% combining this with levetiracetam as an adjunctive ASM. Other ASM were less frequently chosen at this time. The preferred benzodiazepine of choice here was lorazepam in 71.6%. Diazepam and clonazepam were chosen equally in 11.4%, midazolam in 38.3% (multiple benzodiazepines could be chosen) (Figure 1).

The diagnostics chosen at this time (with the possibility of multiple answers) varied between performing a cerebral computer tomography (cCT), cerebral magnetic resonance imaging (cMRI), and cerebrospinal fluid (CSF) diagnostics or no diagnostics at the given time. EEG was reported as being routinely used in 40.24% of cases, whereas long-term (video) EEG monitoring was indicated by only 4.88% of respondents (Table 1). However, the survey did not capture whether EEG facilities were continuously available or restricted to office hours. This limitation prevents a precise evaluation of EEG accessibility across centers.

**Table 1:** Immediate diagnostic procedures after SE onset.

Which diagnostics would you initiate at this point?	
cMRI (basic)	11.0
cMRI (epilepsy programm)	7.3
cCT (native)	47.6
cCT (multimodal)	45.12
CSF	35.4
EEG	40.2
cEEG	4.9
Others	18.3

**Figure 2:**
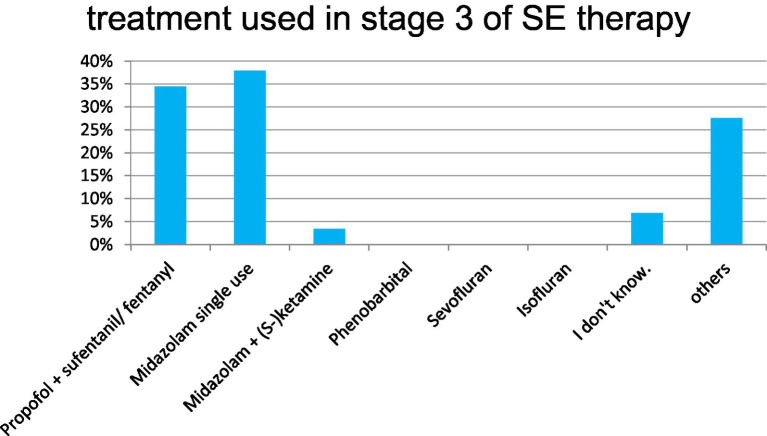
SE therapy, stage 3 (drug escalation).

Next, the scenario evolved describing an established convulsive SE. In this setting, 35.4% of the respondents prefer iv-levetiracetam (after prior single administration of benzodiazepines), 13.4% chose lacosamide, and 31.7% had chosen immediate anesthesia. The drug of choice here was propofol monotherapy in 25%, in combination with sufentanil/ fentanyl in 43.2% (Figure 2).

**Figure 3:**
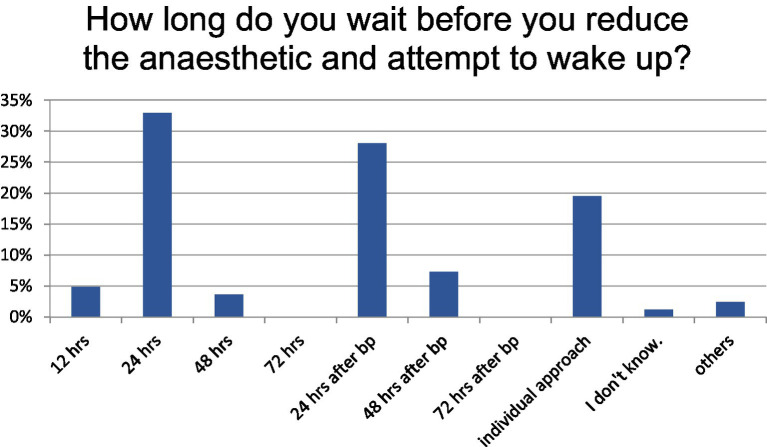
Duration of anesthetic approach, hrs, hours; bp, achieving burst suppression.

At the decision point for initiating anesthesia in the case-based scenario, anesthesia was induced with propofol combined with sufentanil or fentanyl in 65.43% of cases, and with propofol monotherapy in 18.5%. The intended duration of anesthesia varied: 32.9% targeted a fixed 24-h period, 28.1% continued for 24 h following the onset of burst suppression, and 19.5% individualized the duration based on clinical assessment (Figure 3).

If SE persisted and progressed to SRSE, 11% of the respondents opted for a wait-and-see approach, 35% changed the anesthetic agent (with 36% changing PRO to MDZ with S-ketamine, and 19% to a barbiturate.), and 41% escalated treatment by adding an additional ASM.

If additional ASM chosen for escalated treatment at this stage:

**Table:** 

Valproic acid (VPA)	67.11%
Lacosamide (LCM)	64.47%
Levetiracetam (LEV)	43.42%
Perampanel (PER)	38.16%
Phenytoin (PHT)	34.21%*
Brivaracetam (BRV)	21.05%
Phenobarbital (PB)	17.11%
Topiramate (TPM)	15.79%
Zonisamide (ZNS)	7.89%
Cenobamate (CNB)	3.95%
Stiripentol (STP)	2.63%
Lamotrigine (LTG)	2.63%
Cannabidiol (CBD)	1.32%
Bromide (BR)	0.00%

*The survey question did not differentiate between phenytoin and fosphenytoin, as fosphenytoin is approved in Germany and Austria but not marketed, and is not approved in Switzerland. Thus, the reported 34.2% “phenytoin” use in SRSE may have included fosphenytoin, but the authors assume that phenytoin was administered.

Treatment sequencing varied widely. Levetiracetam, lacosamide, and valproic acid were most frequently selected among the first three ASM, although no consistent preference emerged regarding the order. Not all respondents provided dosage information. However, where available, these were within the ranges recommended by the guideline.

Occasionally, stage 3 was also initiated after the administration of a single stage 2 medication.

Similarly, no clear consensus was observed regarding the use of adjunctive therapies such as ketogenic diet or steroids. Beyond the administration of ASM, additional therapeutic strategies were also considered.

These are used in clinical practice with the following frequency:

**Table:** 

Ketogenic diet	49.4%
Corticosteroid pulse therapy	44.8%
Electroconvulsive therapy (ECT)	11.9%

Targeted temperature management (TTM)/hypothermia, deep brain stimulation, transcranial magnetic stimulation (TMS), plasmapheresis and administration of intravenous immunoglobulins (IVIG) were sporadically mentioned.

### Case 2

Case 2 described a 63-year-old woman with structural epilepsy treated with 1,500 mg levetiracetam daily, who presented to the emergency department with generalized convulsive status epilepticus. Her last seizure had occurred 6 months ago, prompting levetiracetam dose escalation from 1,000 to 1,500 mg.

This clinical information would not change the therapeutic approach for 65.2% of the respondents.

## Part 2: prehospital management and diagnostic procedures

### Prehospital management

Prehospital management is critical for the early recognition of SE. The question of whether SE is reliably recognized prehospitally, 74.2% of the respondents indicated that convulsive SE is reliably identified, whereas non-convulsive SE often is not. A standard operating procedure (SOP) or algorithm for SE management was available to 31.8% of emergency services, while 21.2% reported no access to such protocols, while almost half of the participants were unsure.

The prehospital benzodiazepine administration differed by agent: lorazepam was administered in 9.1%, diazepam in 37.9% and others, predominantly midazolam, in 50%. Doses ranged between 2 and 10 mg midazolam (or equivalent dose). Further, 21.2% of the respondents indicated that an anti-seizure medication was administered preclinically, most commonly levetiracetam.

### Diagnostic procedures

In addition to pharmacological treatment, the survey assessed diagnostic practices in patients with SE: In patients with known epilepsy, 96.9% would order laboratory test (including electrolytes and infection marker), and 69.2% would order a cranial CT (cCT); only 10% reported performing MRI or CSF analysis. In contrast, patients with NORSE were more comprehensively evaluated: all recommended laboratory testing including electrolytes and infection parameters, 70.8% a cCT, 78.5% a cMRI and almost 80% CSF analysis. Although the results of ASM drug levels are rarely available in the emergency department, it is very important that this is done upon admission, as low levels can explain SE and can also be used to improve compliance subsequently. This was not explicitly asked in the survey.

### Use of scores

When used (used by 6% only and by 12% occasionally), the most frequently used score was the STESS (Status epilepticus severity score), while other prognostic scores such as ACD (age at onset, level of consciousness at admission, and duration of status epilepticus), EMSE-EAC (Epidemiology-based Mortality Score in Status Epilepticus—Etiology, Age, level of Consciousness) and END-IT were rarely employed.

In this context, it should be emphasized that the ADAN score proposed by Requena and colleagues (11) is a diagnostic tool designed to support the early recognition of status epilepticus in the emergency medical setting. The ADAN scale was specifically designed for prehospital identification of SE when EEG is not immediately available. It is based on four easily assessable clinical parameters: Abnormal speech, Deviation of gaze (ocular deviation), Automatisms, and Number of seizures. The scale aims to assist EMS and emergency physicians in differentiating SE from other causes of impaired consciousness and to promote earlier recognition and treatment initiation. This tool is, to date, one of the very few diagnostic scores validated for potential use in prehospital and emergency department contexts and does not rely on EEG findings.

## Discussion

The findings of this web-based survey among clinicians in Germany, Austria, and Switzerland highlight substantial variability in the management of SE, particularly in treatment strategies and diagnostic approaches. This observed heterogeneity underscores the lack of standardized consensus in key aspects of SE management and care and highlights the complexity and challenges faced by clinicians in managing this neurologic emergency. The development of evidence-based SE guidelines is particularly difficult in intensive care settings where there is little prospective data and a high degree of heterogeneity, for example due to different aetiologies, and requires extensive registry data. The new European registry coordinated by Prof. Nicolas Gaspard represents a unique opportunity to systematically capture treatment and outcome data across centers (12). Such initiatives are an important step toward fact-based rather than consensus-only guidelines.

### Medication choices and dosage

The survey confirms a strong preference for benzodiazepines as first-line therapy in status epilepticus (SE), with lorazepam selected by 71.6% of respondents—aligning with guideline recommendations (13).

While lorazepam appears favored in hospital settings, its requirement for cooled storage limits its prehospital use. Following initial benzodiazepine administration, 56.6% of clinicians selected levetiracetam as a second-line agent, consistent with growing evidence supporting its role in early SE management (14). In contrast, ASM such as lacosamide (13.4%) were less commonly used, possibly due to lower familiarity, lack of authorization and contraindications in patients with atrioventricular conduction abnormalities (15).

Third-line therapies for refractory status epilepticus showed greater heterogeneity. Propofol, often used in combination with fentanyl or sufentanil, was the most frequently chosen option, aligning with established recommendations for SRSE management (16).

Decisions regarding the duration of anesthesia varied: 35% targeted 24 h after achieving burst suppression, whereas 19.5% individualized treatment duration based on clinical judgment. Current guidelines remain noncommittal regarding whether burst suppression or seizure cessation should guide anesthetic depth, and available evidence does not clearly favor one strategy over the other (17).

These findings underscore the variability in SE management—particularly in third-line therapy—and reflect both the complexity of clinical decision-making in SRSE and the absence of robust, consensus-driven protocols for anesthetic duration (18). Although established ICU outcome scores such as the simplified acute physiology score (SAPS) and the Acute Physiology and Chronic Health Evaluation (APACHE) as well as the Complication Burden Index (CBI) (19) were not included in this survey, their systematic incorporation in future registry-based studies would provide valuable context for comparing treatment outcomes across centers. These scoring systems are used to assess disease severity (SAPS, APACHE) and to quantify the cumulative burden of medical complications during ICU stay (CBI). Including such standardized outcome metrics in large-scale SE registries could facilitate benchmarking between hospitals, improve the understanding of treatment-related morbidity, and support the development of evidence-based prognostic tools for SE management.

Despite adherence to guideline-recommended agents, the lack of standardized treatment sequencing remains evident.

### Prehospital management

The prehospital management remains a critical point in the overall course of SE. A multicenter prospective registry study from Germany, Austria and Switzerland reported that over one-third of SE cases presented initially in the prehospital setting, however, a substantial proportion were treated subtherapeutically inconsistent with established guidelines. The clinical impact of such deviation remains unclear but underscores the need for improved prehospital protocols (9).

The survey revealed that the majority of clinicians (74.2%) felt that convulsive SE was reliably recognized prehospitally, while non-convulsive SE was found frequently missed. This diagnostic gap is concerning, given the potential morbidity associated with NCSE (20, 21). Several diagnostic scoring systems for status epilepticus (SE) have been proposed over the past decade, but few have entered routine clinical use. These include the EMSE-EAC score (37) and the more recent ADAN score, developed specifically for prehospital SE recognition (11). While the EMSE-EAC aims to classify SE subtypes and predict outcomes using EEG, etiology, and comorbidity data, the ADAN score provides an EEG-independent diagnostic tool based purely on clinical parameters. Despite their potential utility, both have seen limited adoption in everyday practice—primarily due to a lack of external validation, complexity of data collection, and limited integration into emergency workflows.

Our findings mirror this gap between research and clinical implementation: most respondents reported not using any structured diagnostic or prognostic scoring system. To bridge this gap, future efforts should focus on validating simplified, time-efficient tools that can be integrated into electronic documentation systems or combined with emerging artificial intelligence based support systems to facilitate rapid and accurate SE diagnosis, especially in prehospital and emergency department settings.

The use of easy to apply, EEG detection systems, potentially supported by artificial intelligence might also offer viable solutions here (22, 23). The availability of EEG, especially in the emergency department and prehospital settings, remains a crucial issue. Continuous EEG is essential for detecting non-convulsive SE and assessing treatment response, as highlighted in recent studies (24, 25). Nevertheless, the lack of round-the-clock EEG coverage in many hospitals and the absence of EEG in most emergency medical services create a diagnostic gap. Early EEG recording—even short, portable or tele-EEG approaches—has been shown to improve early recognition of NCSE and guide timely therapy initiation in emergency settings.

Midazolam was the most frequently administered benzodiazepine in the prehospital setting, in line with current guidelines, (5, 26). However, wide dosing variability (2–10 mg) highlights the lack of standardized administration protocols, and persistent underdosing remains a critical concern (9, 27).

ASMs are often administered preclinically, highlighting concerns about the underdosing of benzodiazepines (8, 27). The use of ASMs prior to hospital arrival was also reported, most commonly levetiracetam. This may reflect inappropriate substitution for first-line benzodiazepines, particularly in elderly patients or those with reduced consciousness, despite a lack of supporting evidence (28, 29). These findings emphasize the need for targeted education, protocol harmonization, and prospective evaluation of prehospital SE interventions to ensure adherence to evidence-based standards.

The limited availability of standard operating procedures (SOPs) for SE management observed in our survey highlights a critical gap in the standardization of emergency care. Only about one-third of respondents reported having access to a defined SE protocol, while almost half were unsure whether such a guideline existed in their institution. The lack of clear, structured algorithms may delay escalation of therapy and contribute to outcome variability between centers. Although our study did not explicitly assess regional differences, anecdotal responses suggest heterogeneity among German-speaking countries, reflecting differences in hospital organization and training structures. The need for unified, evidence-based SOPs extends therefore beyond the German-speaking area. A recent study from Norway highlighted this variability, showing that as many as 18 different emergency protocols for SE were in use within a relatively small country (30). This lack of standardization was associated with delayed treatment escalation and inconsistent use of anesthetics in refractory SE. Developing harmonized European or international protocols—integrating prehospital and in-hospital treatment recommendations—could therefore improve therapeutic consistency and facilitate multicenter research comparability.

The survey did not systematically assess differences between emergency medical service (EMS) structures, which represents a limitation of the present study. However, the existing literature indicates considerable heterogeneity between EMS systems. Benzodiazepines remain the only anti-seizure medication consistently available in most EMS units, serving as the universally recommended first-line treatment for prehospital SE management (31). This has been confirmed in more recent data from the United States, showing that intravenous lorazepam, intravenous or intramuscular diazepam, and intramuscular midazolam are the mainstay of prehospital SE therapy (32).

Across Europe, substantial variability in prehospital SE protocols persists, with inconsistent dosing recommendations, administration routes, and timing.

Second-line ASMs such as levetiracetam or valproate are rarely available in ambulances and are usually administered only after hospital arrival (28, 33). However, several European emergency medical systems—most notably in France and Finland—have integrated the prehospital administration of second-line ASMs, particularly intravenous levetiracetam, into routine practice. The SAMUKeppra study randomised phase 3 trial demonstrated the feasibility and safety of adding levetiracetam to clonazepam in the prehospital treatment of SE (29), and other studies further emphasise that earlier ASM administration and reduced treatment delays are associated with improved outcomes in SE: A study from 2015 found that in children with convulsive status epilepticus (CSE), the administration and escalation of ASM were substantially delayed both before and after hospital arrival, often taking over an hour from seizure onset (34). In addition to that, Kämppi et al. (35) showed that in adults with generalized CSE, treatment delays—especially longer than 2.5 h for diagnosis or escalation of antiseizure therapy—were linked to poorer outcomes, highlighting the need for rapid diagnosis, timely treatment, and prompt transfer to specialized hospitals. These experiences underline that minimising prehospital treatment delays—through protocol adaptation and drug availability—should be a central goal in EMS care for SE.

Overall, these data highlight the wide heterogeneity of prehospital SE treatment capabilities and underscore the urgent need for harmonized EMS protocols and standard operating procedures across regions.

### Diagnostic procedures

The survey showed that the variance in diagnostic procedures was significantly smaller than the variance in treatment, indicating good practice in neurological emergencies. Only the initial application of prognostic scores is highly variable and has seen limited integration into clinical routine, in part because their sensitivity and specificity are insufficient for individual clinical decision-making (29). Better validated scoring systems are needed, and artificial intelligence approaches may help to improve future predictive accuracy.

### Limitations

The study has several limitations. The sample size was small and predominantly composed of respondents from university hospitals with rapid access to neuro-intensive care units and experience on treating neuro-intensive care patients. In addition, the majority of participants were experienced clinicians; young colleagues, who often provide primary therapy in the emergency department, being underrepresented. Despite the bias in favor of highly experienced respondents, heterogeneity in treatment was reported. The clinical scenarios focused specifically on the management of generalized convulsive SE. It might occur that treatment choices would differ in other contexts, such as focal motor SE or NCSE. However, given that treatment guidelines focus on generalized convulsive SE, the observed heterogeneity underscores the significant variability in clinical practice, even within standardized scenarios.

## Conclusion

This survey highlights current practices in the management of SE in the German-speaking countries of Europe. With lorazepam and midazolam being preferred in intra-hospital and pre-hospital settings. From stage 2 of treatment onwards, there are trends, such as the use of levetiracetam for stage 2 and propofol for stage 3. Management of SRSE remains highly variable due to limited guideline evidence. Standardization of prehospital treatment, ICU diagnostics and therapy monitoring is needed.

## Data Availability

The raw data supporting the conclusions of this article will be made available by the authors, without undue reservation.

## Group members of IGNITE Group and the MuSE Group

IGNITE: Initiative of German Neurointensive Trial Engagement, a Section for Clinical Studies within the German Society for Neurointensive and Emergency Care (Deutsche Gesellschaft für NeuroIntensiv- und Notfallmedizin, DGNI). IGNITE Group members are listed in the Supplementary material.

MuSE: multicentric Studies in Epilepsy, part of the German Society for Epilepsy (Deutsche Gesellschaft für Epileptologie, DGfE), Austrian Society for Epilepsy (Österreichische Gesellschaft für Epileptologie, ÖGfE) and Swiss Epilepsy League (Schweizerische Epilepsie-Liga).
