# Supplementary material for: Treating status epilepticus in clinical practice—a multi-national survey in Germany, Austria, and Switzerland
Source: Front Neurol. 2025 Nov 3;16:1685993. doi: 10.3389/fneur.2025.1685993 (PMC12620260; doi:10.3389/fneur.2025.1685993)
Supplement: Supplementary file 1 [file Data_Sheet_1.DOCX]

**Supplementary material**

**INFORMATION**
The aim of this study is to gain a better understanding of how patients with status epilepticus are treated in routine clinical practice in Germany, Austria, and Switzerland, both in prehospital and in-hospital settings.
We kindly ask you to complete this questionnaire. The questionnaire is case-based. The clinical cases described have occurred in a similar or modified form in our clinical practice; however, no conclusions can be drawn about a specific patient.
Your decision to participate is voluntary. You may decline to answer any questions at any time. However, please note that data from partially completed questionnaires may still be used.
Completing the questionnaire should take approximately 15 minutes.

If you have any questions about this study, please contact:

| **Dr Leona Möller, MHBA** Epilepsy Centre Hessen Philipps-University Marburg Phone: +49 6421 58 65435 Email: leona.moeller@med.uni-marburg.de | **Dr Dr Urs Fisch** University Hospital Basel Email: urs.fisch@usb.ch |
| --- | --- |

**CASE 1**

**Question 1:**
A 36-year-old patient (80 kg) is presented to the emergency department by ambulance staff. He received 5 mg of diazepam preclinically for a generalised convulsive status epilepticus (SE), which failed to terminate the SE. What would be your next step?

- Repeat administration of benzodiazepines
- Immediate administration of levetiracetam
- Immediate administration of valproate
- Immediate administration of lacosamide
- Immediate administration of phenytoin
- Immediate anaesthetic intubation
- No further therapy at this stage – initiate diagnostic procedures first

**Question 2:**
Which benzodiazepine do you use?

- Lorazepam
- Diazepam
- Clonazepam
- Midazolam
- I don't know
- Other

**Question 3:**
If you selected “immediate anaesthetic intubation”: Which medication would you use in this case? [Multiple answers possible]

- Propofol monotherapy
- Propofol + sufentanil/fentanyl
- Midazolam monotherapy
- Midazolam + (S)-ketamine
- Phenobarbital
- Sevoflurane
- Isoflurane
- I don't know
- Other

**Question 4:**
At what dose would you administer this medication? [Please specify in mg]

**Question 5:**
What diagnostic procedures would you initiate at this point? [Multiple answers possible]

- cMRI (basic)
- cMRI (epilepsy protocol)
- cCT (non-contrast)
- cCT (multimodal)
- CSF examination
- EEG
- cEEG
- Other

**Question 6:**
Fifteen minutes after your previous treatment, the SE persists. What is your next step?

- Repeat administration of benzodiazepines
- Immediate administration of levetiracetam
- Immediate administration of valproate
- Immediate administration of lacosamide
- Immediate administration of phenytoin
- Immediate anaesthetic intubation
- Other

**Question 7:**
If you selected “repeat administration of benzodiazepines”: Which benzodiazepine would you use in this case? [Multiple answers possible]

- Lorazepam
- Diazepam
- Clonazepam
- Midazolam
- I don't know
- Other (please specify)

**Question 8:**
If you selected “anaesthetic intubation”: Which medication would you use in this case? [Multiple answers possible]

- Propofol monotherapy
- Propofol + sufentanil/fentanyl
- Midazolam monotherapy
- Midazolam + (S)-ketamine
- Phenobarbital
- Sevoflurane
- Isoflurane
- I don't know
- Other (please specify)

**Question 9:**
At what dose would you administer this medication? [Please specify in mg]

**Question 10:**
The SE could not be terminated, making ICU admission and rapid intubation necessary. Which of the following medications is typically used in your hospital for anaesthesia during SE treatment?

- Propofol monotherapy
- Propofol + sufentanil/fentanyl
- Midazolam monotherapy
- Midazolam + (S)-ketamine
- Phenobarbital
- Sevoflurane
- Isoflurane
- Other (please specify)

**Question 11:**
How long do you wait before reducing sedation and attempting to wake the patient?

- 12 hours
- 24 hours
- 48 hours
- 72 hours
- 24 hours after burst suppression
- 48 hours after burst suppression
- 72 hours after burst suppression
- Individual approach
- I don't know
- Other

**Question 12:**
If the status still does not terminate, what is your next step?

- Repeat sedation with the same medication
- Repeat sedation with a different medication
- Additional anti-seizure medications
- Further treatment options
- I don't know
- Other (please specify)

**Question 13:**
If you selected “repeat sedation with a different medication”: Which medication would you choose? [Multiple answers possible]

- Propofol monotherapy
- Propofol + sufentanil/fentanyl
- Midazolam monotherapy
- Midazolam + S-ketamine
- Phenobarbital
- Sevoflurane
- Isoflurane
- I don't know
- Other (please specify)

**Question 14:**
If you selected “additional anti-seizure medications”: Which would you choose? [Multiple answers possible]

- Valproate (VPA)
- Lacosamide (LCM)
- Levetiracetam (LEV)
- Perampanel (PER)
- Phenytoin (PHT)
- Brivaracetam (BRV)
- Phenobarbital (PB)
- Topiramate (TPM)
- Zonisamide (ZNS)
- Cenobamate (CNB)
- Stiripentol (STP)
- Lamotrigine (LTG)
- Cannabidiol (CBD)
- Other

**Question 15:**
If you selected “further treatment options”: Which would you use? [Multiple answers possible]

- None of the above
- Steroid pulse therapy
- Ketogenic diet
- Electroconvulsive therapy (ECT)
- I don't know
- Other (please specify)

**Question 16:**
If you selected multiple options: Please specify the order in which you would usually administer the therapies:

**CASE 2**
In the emergency department, a 63-year-old female patient with known structural epilepsy and anti-seizure medication (1500 mg levetiracetam daily dose) is presented. She has been taking her medication reliably. According to third-party history, her last seizure occurred 6 months ago, after which the levetiracetam dose was already increased. She is currently experiencing a generalised status epilepticus.

**Question 17:**
Does your primary approach differ compared to Case 1?

- No
- Yes

**Question 18:**
If yes: What would your approach be now?

- No administration of benzodiazepines
- No additional administration of levetiracetam
- Immediate administration of lacosamide
- Immediate administration of valproate
- Other (please specify)

**Question 19:**
If you determine that the patient has not taken her medication in the past 2 days, would this change your approach?

- Yes
- No
- I don't know

**Question 20:**
If yes: What would your approach be now?

- Immediate administration of intravenous levetiracetam
- Immediate administration of intravenous brivaracetam
- Other (please specify)

**PREHOSPITAL MANAGEMENT**

**Question 21:**
Do you believe that status epilepticus (SE) is reliably recognised in the prehospital setting?

- Yes, almost all cases
- Yes, but not non-convulsive SE
- Rather not
- No
- I don't know
- Other (please specify)

**Question 22:**
Does your emergency service have a treatment guideline/SOP/algorithm for managing SE?

- Yes
- No
- I don't know

**Question 23:**
Are benzodiazepines administered prehospitally by emergency service personnel?

- Yes, lorazepam
- Yes, diazepam
- Yes, other
- No
- I don't know

**Question 24:**
At what dose is this medication typically administered? [Please indicate in mg]

**Question 25:**
Are anti-seizure medications administered prehospitally by emergency service personnel?

- Yes
- No
- I don't know

**Question 26:**
Which anti-seizure medication is administered?

**DIAGNOSTICS**

**Question 27:**
Which diagnostics do you routinely initiate in patients with known epilepsy and ongoing SE? [Multiple answers possible]

- None, unless there are signs of infection/fall, etc.
- Blood sampling including electrolytes and infection markers
- In addition to “basic blood work”, prolactin and further specialised laboratory diagnostics if necessary
- Cranial CT (cCT)
- Cranial MRI (cMRI)
- Basic CSF diagnostics
- CSF diagnostics including autoimmune panel
- Other (please specify)

**Question 28:**
Which diagnostics do you routinely initiate in patients with new-onset SE? [Multiple answers possible]

- Blood sampling including electrolytes and infection markers
- In addition to “basic blood work”, prolactin and further specialised laboratory diagnostics if necessary
- Cranial CT (cCT)
- Cranial MRI (cMRI)
- Basic CSF diagnostics
- CSF diagnostics including autoimmune panel
- Other (please specify)

**Question 29:**
Do you routinely use SE-related scores for documentation/diagnostics?

- Yes
- No
- Rarely
- I don't know

**Question 30:**
If yes, which ones? [Multiple answers possible]

- STESS (Status Epilepticus Severity Score)
- QOLIE (Quality of Life in Epilepsy)
- ACD score
- EMSE-EAC (Epidemiology-based Mortality Score in Status Epilepticus - Aetiology, Age, level of Consciousness)
- ENDIT (Encephalitis, non-convulsive status epilepticus, Diazepam resistance, Imaging abnormalities, Tracheal intubation)
- I don't know
- Other (please specify)

**GENERAL INFORMATION**

To conclude, we would like to ask you a few questions about yourself:

**Question 31:**
What is your level of training?

- Junior doctor
- Specialist
- Senior consultant
- Head of department
- Other (please specify)

**Question 32:**
Do you hold any of the following additional qualifications? [Multiple answers possible]

- None of the above
- Intensive care medicine
- Epileptology
- EEG certification
- Paediatric neurology
- Emergency medicine
- Other (please specify)

**Question 33:**
In which area are you employed? [Multiple answers possible]

- Outpatient sector
- Tertiary care hospital
- University hospital
- Epilepsy centre
- Primary or secondary care hospital
- Other (please specify)

**Question 34:**
Please indicate the first digit of your postal code area:

- 0
- 1
- 2
- 3
- 4
- 5
- 6
- 7
- 8
- 9

**Thank you very much for participating in this study!**
